# Providing palliative care for residents in LTC facilities: an analysis of routine data of LTC facilities in Lower Saxony, Germany

**DOI:** 10.1186/s12904-022-00998-1

**Published:** 2022-06-23

**Authors:** Wenke Walther, Gabriele Müller-Mundt, Birgitt Wiese, Nils Schneider, Stephanie Stiel

**Affiliations:** Institute for General Practice and Palliative Care, Medical School Hannover, Carl-Neuberg-Str. 1, 30625 Hannover, Germany

**Keywords:** Palliative care, Nursing homes, Diseased residents, Nursing records, Advance care planning, Hospitalization

## Abstract

**Background:**

Demographic trends show an increasing number of elderly people and thus a growing need for palliative care (PC). Such care is increasingly being provided by long-term care (LTC) facilities. The present study aimed at exploring PC indicators of residents at LTC facilities belonging to a non-profit provider in Lower Saxony, Germany, in order to identify potential improvements.

**Methods:**

A descriptive cross-sectional study was conducted, drawing on routine nursing chart data. Structural data from 16 participating LTC facilities and the care data of all residents who died in 2019 (*N* = 471) were collected anonymously between March and May 2020. Based on key literature on quality indicators of PC in LTC facilities in Germany, a structured survey was developed by a multidisciplinary research team. The descriptive, comparative and inferential data analysis was conducted using the SPSS software package.

**Results:**

In total, the complete records of 363 (77%) residents who died in the participating LTC facilities in 2019 were retrieved. The records reflected that 45% of the residents had been hospitalized at least once during the last 6 months of their lives, and 19% had died in hospital. Advance care planning (ACP) consultation was offered to 168 (46%) residents, and 64 (38%) declined this offer. A written advance directive was available for 47% of the residents. A specialized PC team and hospice service volunteers were involved in caring for 6% and 14% of the residents, respectively. Cancer patients received support from external services significantly more frequently (*p* < .001) than did non-cancer patients. Differences emerged in the distribution of PC indicators between LTC facilities. Facilities that have more PC trained staff offered more ACP, supported by more specialized PC teams and hospice services, and had fewer hospitalizations. In addition, more volunteer hospice services were offered in urban facilities.

**Conclusions:**

Overall, a rather positive picture of PC in participating LTC facilities in Germany emerged, although there were differences in the expression of certain indicators between facilities. ACP consultation, volunteer hospice services, and hospital admissions appeared to be superior in LTC facilities with more trained PC staff. Therefore, PC training for staff should be further promoted.

## Background

Due to the ongoing sociodemographic transition the number of older and chronically ill people in the global population increased [1], and thus the demand for care in the last phase of life [2]. Such care, which typically involves some degree of palliative care (PC), is often provided within a long-term care (LTC) facility. Over the past decade, patients in Germany have begun to move into LTC facilities at an older age and, accordingly, spend less time there [3]. Therefore, regularly collected care statistics in Germany show that the proportion of LTC facility residents requiring care has increased in recent years [4]. Residents are typically suffering from a variety of chronic and progressive illnesses [5].

In the German federal state of Lower Saxony, approximately 112,000 people are currently accommodated in LTC facilities [4], where they will probably also die. In the region of Hannover, for example, the proportion of all deceased citizens who died in a LTC facility was 27% in 2017, compared to 15% in 2007 [6]. Thus, the need for PC for residents at the end of life in LTC facilities is increasing. The PC approach is understood as one that is comprehensive and interdisciplinary, respecting patients’ wishes and needs for the end of life. It aims at addressing both physical and psychological symptoms, as well as social and spiritual issues, and it attempts to fulfil patients’ wish. In order to improve the structured continuous recording of end-of-life wishes (ACP) of residents, agreements were reached with the health insurers in Germany in December 2017 on the basis of the law § 132 g part 3 SGB V, which regulates the implementation of ACP counseling by extensively special trained ACP counselors [7]. Several studies have shown that many people wish to die in a familiar environment [8], without pain and in the company of others [9]. In addition, PC seeks to avoid unnecessarily life-prolonging or burdensome treatments, such as artificial nutrition, as well as frequent hospital admissions [10]. Therefore, the early recognition of PC needs is essential [11]. To meet the growing requirements for PC in Germany, generalist PC offerings in LTC facilities must be strengthened. For this purpose, external specialized PC services for residents with complex needs must also be integrated at an early stage. However, the transfer of specialized PC to the LTC system seems to be challenging. According to previous research, LTC facilities typically lack personnel, skills, knowledge, and time for PC [11, 12].

Various initiatives have been undertaken, both nationally and internationally, to establish a hospice culture and to improve the administration of PC in LTC facilities [13, 14]. In particular, some non-profit providers of LTC facilities in Germany have conducted model projects for the implementation of PC (for an overview, see [14], p.36 ff). In 2006, a non-profit provider in Lower Saxony launched an initiative to strengthen hospice culture and PC competence within its LTC facilities. Since 2013, more than 30 PC trainings of 40 h were offered to staff in all areas (nursing, companionship, and housekeeping) for this purpose. Until 2018, a total of 582 employees in 75 facilities had been reached in Lower Saxony. The present study contributed to the ImPAct project (Implementation of palliative competence and hospice culture in LTC facilities) which aims at developing recommendations for high-quality PC in LTC facilities. We use merged data from all LTC facilities, drawing on multiple perspectives and databases, including nursing records of deceased residents. Therefore, this analysis can be understood as an independent status quo report on all facilities of the provider.

This paper presents the results of an analysis of the nursing chart data of residents who died in 2019, with respect to PC in the last phase of life. It concludes by identifying potential improvements for PC within LTC facilities in Germany.

## Methods

### Study design and data collection

A retrospective analysis of the routine nursing chart data of the last 12 and 6 months of life of LTC facility residents who died in 2019 was conducted. All 146 LTC facilities in Lower Saxony of a particular protestant non-profit provider were invited by post to participate in the ImPAct study in December 2019, and to nominate one contact person. After 2 weeks, all facilities were asked again about their willingness to participate, via telephone. In January 2020, 18 (12%) LTC facilities agreed to participate in the study. Main reasons stated for not-participation were limited resources (38%) and involvement in other projects (33%). General structural data for the facilities and the number of deceased residents in 2019 were requested. In a second step, 491 (corresponding to the data gathered in the previous step) anonymous survey forms were sent out to the contact persons at the participating LTC facilities. These persons were invited to fill in one survey for each deceased resident (data collection period: March to May 2020). A reminder letter was sent to the participating LTC facilities in April 2020.

For the anonymized data collection, the contact persons in the participating LTC facilities were asked to extract certain data into a standardized survey form. Based on key literature on quality indicators for PC in LTC facilities [8, 15–18] and as far as documented in the nursing record, this one-page data sheet was developed by a multidisciplinary research team. Covered are structural quality parameters that indicate the availability of palliative care providers (specialized PC, volunteer hospice service) and process and result quality parameters that indicate the identification, documentation, and respect of residents’ wishes (ACP consultation, advance care documents, DNR-order, adherence to them, hospital admissions, place of death). Personal data and facility data were supplemented. Reviews showed that PC is more effectively provided in larger urban facilities [19] among women and residents with cancer [17, 20, 21]. Similarly, there is evidence that family members influence PC, such as hospitalizations, companionship, and ACP [22–24]. Also, studies showed that there is evidence that resident age and PC training may influence PC [25–27] (Table 1).

**Table 1:** Data pertaining to general facility structure and select PC indicators, collected from the nursing charts of deceased residents

	**Collected data**
**Structural data**	
Facility	Region
	Number of beds
Residents	Mean age at admission, in years
	Age structure (number of residents < 60, 60–69, 70–79, 80–89,90–99, > 100 years)
	Sex
	Care level, according to the German compulsory nursing insurance scheme^a^
Staff	Number of staff
	Number of staff with
	• Basic palliative care training (40 h)
	• Advanced palliative care training (160 h)^b^
**Nursing chart data of residents who died in 2019**	
Personal data	Age
	Sex
	Date of admission
	Date of death
	Main diagnosis/diagnoses
	Care grade, according to the German compulsory nursing care insurance scheme
	Next of kin (known to the facility)
PC indicators	Specialized PC (yes/no; if yes, how long, in weeks?)
	Voluntary hospice service (yes/no; if yes, how long, in weeks?)
	Admission to hospital in the last 6 month of life (number of admissions, duration)
	Advance care planning offer (yes/no/yes, but offer rejected)^c^
	Health care proxy (in written form, yes/no)
	Advance directive (in written form, yes/no)
	if yes: do not resuscitate order (yes/no; if yes: this request was complied: yes/no)

^a^This insurance covers the risk of becoming dependent on nursing care and attention, which may arise as a result of a serious accident, disease or old age. Nursing care insurance is obligatory for everyone with health insurance. Need for care and financing are determined via care grades, as follows: care grade 1: low level of impaired independence or capabilities; care grade 2: significant level of impaired independence or capabilities; care grade 3: serious level of impaired independence or capabilities; care grade 4: the most severe level of impaired independence or capabilities; and care grade 5: the most severe level of impaired independence or capabilities, alongside special long-term care requirements

^b^In Germany there are various trainings for palliative care competencies for LTC facility employees. A certified basic course of 40 h for all staff members and an add-on course of 160 h specifically for nurses[28]

^c^According to law § 132 g part 3 SGB V

### Statistical analyses

Quantitative data were analyzed descriptively and correlations between nominal and ordinal data were calculated with rank correlation coefficient Spearmann roh (r_s_). Group differences for metric data were calculated using T test and for ordinal data using Mann–Whitney U test. To evaluate the impact of facility (region, size) and resident characteristics (age, sex, dementia, cancer, length of stay) on PC variables (integration of specialized palliative care, voluntary hospice service, hospital admission, place of death) linear mixed effects regression models were applied. To take into account the cluster structure, the LTC facilities were included as random effects in the model. Data were analyzed using the Statistical Package for Sciences, version 26 (SPSS Inc., Chicago, IL, USA) and STATA, version 16.

## Results

Of the 18 LTC facilities who provided contact persons, only 16 provided data. Across these 16 LTC facilities, 471 residents were recorded as having died in 2019. Until April 2020 we received 308 (65%) data forms on the deceased residents. After the reminder letter additional 55 (12%) data forms were returned, so that in total 363 data forms were send back completely until May 2020 (77%).

### Characteristics of the LTC facilities

The majority of the 16 LTC facilities were located in small- to mid-sized cities (Table 2). On average, they had 86 (min.-max.: 40–160) care spaces and 62 (min.-max.: 35–128) employees, of whom approximately 60% were nurses. Each facility had an average of 2.5 employees (min–max: 0–6) with advanced PC training (160 h) and 7.5 employees (min–max: 0–47) with basic PC training (40 h). Only in one facility, none of the employees had a PC training.

**Table 2:** Characteristics of the participating LTC facilities (*n* = 16)

**Characteristics**	
**Facility location**	
Rural (population < 5,000)	2 (12.5%)
Small city (population 5,000–20,000)	6 (37.5%)
Mid-sized city (population 20,000–100,000)	5 (31.3%)
Large city (population ≥ 100,000)	3 (18.8%)
**Facility size**	
Small (< 50 beds)	1 (6.3%)
Mid-sized (50–100 beds)	12 (75.6%)
Large (> 100 beds)	3 (18.9%)
Mean (SD) number of beds	89.5 (27)

According to the information provided by the LTC facility contact persons, the majority of the residents were female (68%), and residents’ average age at the time of admission was 83 years. The facilities mainly accommodated residents on care grades 3 (31%), 4 (30%), 2 (22%), 5 (16%) and 1 (1%), according to the German compulsory nursing care insurance scheme. In 2019, 33% of the residents in these facilities died.

### Characteristics of the deceased residents

Nursing data on the 363 deceased residents revealed that the majority (71%) were female, and their average age was 87 years (SD = 8) at the time of death (Table 3). The age of death of the female residents was significantly higher (M = 88 years; SD = 8) than that of the male residents (M = 84 years; SD = 9; t-test: *p* < 0.001). In total, 333 (92%) of the deceased residents had received regular visits from their next of kin (mainly their children (73%) and spouse (20%)) during their last 6 months of life.

**Table 3:** Characteristics of the residents who died in 2019 (*n* = 363)

**Characteristics**	
**Sex:** female	256 (70.7%)
**Age:** mean (SD) age, in years, at the time of death	87.0 (8.4)
**Length of stay**	
< 1 month	46 (12.7%)
1–6 months	53 (14.6%)
6 months–1 year	31 (8.5%)
> 1 year	229 (63.1%)
Missing value	4 (1.1%)
**Number of chronic diseases:** mean (SD)	2.98 (1.41)
**Number of residents with multimorbidity (three or more chronic diseases)**	221 (62.2%)
**Most frequent diseases at the time of death**	
Heart disease	152 (43.3%)
Dementia	122 (36.8%)
Hypertension	115 (32.8%)
Renal disease	82 (23.4%)
Diabetes	71 (20.2%)
Cerebrovascular disease	68 (19.4%)
Cancer	54 (16.2%)
Chronic obstructive pulmonary disease	41 (11.7%)
Parkinson’s disease	33 (9.4%)
**Care grade, according to the German compulsory nursing care insurance scheme, at the time of death**	
Grade 1	0
Grade 2	38 (10.5%)
Grade 3	68 (18.7%)
Grade 4	121 (33.3%)
Grade 5	130 (35.8%)
Missing values	6 (1.7%)

While nearly two-thirds of the deceased residents had lived in the LTC facility for more than 1 year, 28% had died within their first 6 months in the facility (Table 3). A second sex difference emerged in this respect, with female residents having lived in the facility for an average of 42 months (SD = 54) and male residents having lived there for an average of 25 months (SD = 31; t-test: *p* < 0.001).

Overall, almost two-thirds of the deceased residents had suffered from multiple illnesses (three or more chronic diseases). Furthermore, the majority had been highly dependent on support in their activities of daily living during their last phase of life. At the time of death, 36% of the residents were receiving care level 5, according to the German compulsory nursing care insurance scheme (Table 3).

### Care in the last phase of life

Table 4 displays the results of the descriptive analyses of the selected indicators of care during residents’ last phase of life in the LTC facilities. In this phase, 6% and 14% of the deceased residents received specialized PC and volunteer hospice services, respectively. The duration of these services averaged 5 and 4 weeks, respectively (Table 4). Residents with cancer were significantly more likely to have received both specialized PC and volunteer hospice services compared to residents without cancer (Mann–Whitney U test: *p* < 0.000) (Table 4). Furthermore, there was a weak but significant correlation between residents who received volunteer hospice services and residents with no relatives known to the facility (r_s_: -0.129*).

**Table 4:** End of life care for residents who died in 2019 (*n* = 363)

**Nursing chart data**	
Specialized palliative care	23 (6.4%)
Mean (SD^a^) duration, in weeks	5.1 (6.9)
For residents with cancer (*n* = 57)	9 (15.8%)
Voluntary hospice services (VHS)	50 (13.9%)
Mean (SD^a^) duration, in weeks	4.1 (3.8)
For residents with cancer (*n* = 57)	17 (29.8%)
Residents with a minimum of one hospitalization during their last 6 months of life	163 (44.9%)
Mean (SD^a^) number of hospitalizations	0.6 (0.7)
Mean (SD^a^) days of treatment	7.7 (6.5)
**Advance care planning**	
Offer of advance care planning consultation	168 (46.3%)
Health care proxy (written)	235 (64.7%)
Advance directive (written)	169 (46.6%)
Do not resuscitate order	155 (42.7%)
Complied with	149 (96.1%)
**Place of death**	
Long-term care facility	280 (77.1%)
Hospital	73 (20.1%)
Missing values	10 (2.8%)

^a^*SD* Standard deviation

With respect to hospitalization, 45% of residents were admitted to hospital at least once during their last 6 months of life, with an average length of stay of 8 days. Overall, 20% of the residents living in LTC facilities, died in hospital. Advance care planning (ACP) consultation was offered to 168 (46%) residents. Of these, 64 (38%) refused the offer. Health care proxies and advance directives were registered for 235 (65%) and 169 (47%) residents, respectively. Finally, 43% of the residents had a do not resuscitate (DNR) order as far as it had been documented in their nursing record, which was complied with in 96% of the cases, according to the LTC facility contact persons.

Residents who expressed desires concerning end-of-life care were more likely to die in the facility (r_s_: -0.178**). There was also a weak relationship between the place of death (in the facility) and support administered from voluntary hospice services (r_s_: 0.129*). Finally, there were weak to moderate correlations between facilities’ number of qualified PC staff and: (1) support administered by specialized PC teams (r_s_: 0.123*), (2) support administered by volunteer hospice services (r_s_: 0.109*), (3) number of hospitalizations (r_s_: -0.155**) and (4) ACP consultation offers (r_s_: 0.536**).

**Fig. 1:**
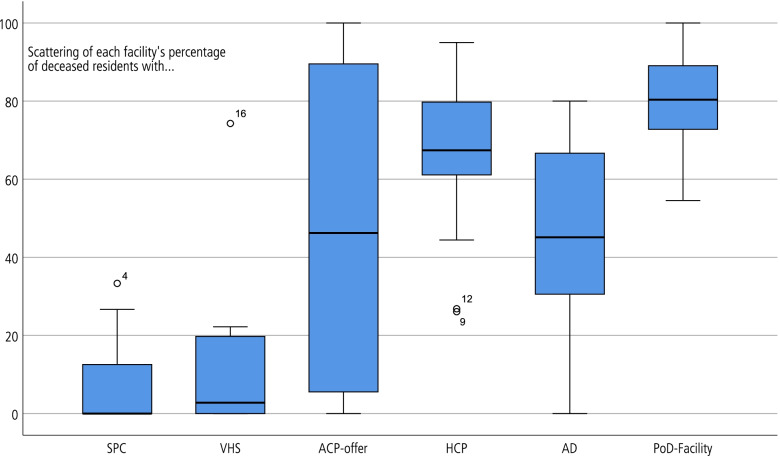
Distribution of indicators for each LTC facility (*n* = 16). (SPC = specialized palliative care, VHS = voluntary hospice services, ACP-offer = offering consultation to advance care planning, HCP = health care proxy, AD = advance directive, PoD = place of death in the facility)

Separate analyses of the PC indicators for each facility revealed significant differences between facilities. For example, in some of the observed facilities, residents never received any volunteer hospice service, while in others, such services were accessed by up to 74% of the residents. Also, some facilities never offered ACP consultation, while others offered ACP consultation to each resident (see Fig. 1).

Analyses were run to test whether selected LTC facility (i.e. region, size) and resident characteristics (i.e. age, sex, length of stay, diagnosis: cancer/dementia) influenced PC delivery. The mixed effects logistic regression (Table 5) showed that LTC facility location had a significant influence on the integration of volunteer hospice services, whereby the likelihood of residents receiving volunteer hospice services was much higher in urban relative to rural areas (odds ratio: 74.75***). Furthermore, residents’ age at the time of death was significantly correlated with hospitalization patterns and death in hospital (odds ratio: 0.97** and 0.96**), whereby hospitalization and death in hospital were less frequently observed in older residents. Sex was also found to exert a significant influence, whereby female residents were more likely to have received voluntary hospice services than were male residents (odds ratio: 5.26***). Additionally, diagnosis of cancer was found to significantly influence whether a patient received specialized PC and volunteer hospice services (odds ratio: 5.20**, odds ratio: 3.97**), whereby patients with a cancer diagnosis were approximately 5 and almost 4 times more likely to receive specialized PC and volunteer hospice services, respectively. Finally, length of stay in the LTC facility had a significant influence on hospitalization, whereby residents who had lived in the facility for more than 1 year were hospitalized 2.6 times more frequently than residents who had lived in the facility for less than 1 year (odds ratio: 2.56**). No other significant correlations were found between the other parameters.

**Table 5:** Analysis of the influence on PC indicators (mixed effects logistic regression)

	**Odds ratio** **p >|z|**			
	**SPC**	**VHS**	**HA**	**PoD**
Region of LTC facility:				
Urban vs. rural	1.725	**74.754**	.339	2.115
	0.792	**0.001**	0.103	0.216
Size of LTC facility:				
Number of beds	1.015	.964	1.011	.990
	0.590	0.183	0.215	0.185
Age of the deceased resident	.998	1.017	**.966**	**.962**
	0.949	0.537	**0.013**	**0.024**
Sex of the deceased resident:				
Female vs. male	1.389	**5.259**	.783	.706
	0.619	**0.005**	0.348	0.278
Disease(s):				
Dementia Yes vs. no	1.015	.964	.803	.710
	0.979	0.938	0.386	0.293
Cancer Yes vs. no	**5.197**	**3.975**	.951	.7640
	**0.004**	**0.011**	0.879	0.519
Length of stay:				
> 1 year vs. < 1 year	2.477	1.587	**2.563**	1.003
	0.450	0.572	**0.025**	0.994

*HA* Hospital admission, *LTC* Long-term care, *PoD* Place of death in the facility, *SPC* Specialized palliative care, *VHS* Voluntary hospice services

## Discussion

The present study aimed at exploring the current status of PC in LTC facilities of a non-profit provider in Lower Saxony, Germany, drawing on routine nursing care data for 363 residents who died in 2019. In summary, 45% of the residents were hospitalized in the last 6 months of life, and 20% died in hospital. 46% and 47% of residents received ACP and had an AD, respectively. 6% received specialized palliative care and 14% voluntary hospice companions.

Overall, a quite positive picture of PC emerged, though the implementation of PC varied widely across the included facilities. Health insurance data show that 70% of deceased residents in German LTC facilities had at least one hospitalization in the 6 months prior to death [29]. In our data, the proportion is considerably lower (45%). Especially at the end of life, hospitalization is particularly stressful for LTC residents and their relatives, and often associated with a deteriorated quality of life [20, 21]. At the same time, not all end-of-life hospitalization are not necessarily inappropriate, especially in emergency situations, and in accordance with the patient’s wishes [22]. In our analysis, younger residents had a higher risk of hospitalization in the last 6 months of their lives. Reviews on the influence of age and sex on emergency department visits and hospitalizations in nursing home residents have not identified a clear trend toward age and found no reasons why younger residents have more hospitalizations [26, 27]. Hypothetically, this may be due to unexpected and sudden deterioration and the assumption that younger residents have a greater chance of recovery.

Few residents (20%) in the current sample died in hospital. Hoffmann and Allers [29] showed that, in the period 2010 to 2014, approximately 30% of nursing home residents died in hospital, each year. Recent data (2016) from a retrospective analysis of 30 LTC facilities in a city in Lower Saxony indicate that 46% of residents died in hospital [30]. Other studies have shown that the majority of residents regard LTC facilities as the environment in which they would like to die [8]. From the present results, we assume that residents’ values and preferences for care and treatment were respected.

The legislated agreement to determine residents’ wishes in terms of ACP (GVP, § 132 SGB V) by special trained ACP consultants has just been implemented in Germany since 2018. Our data show that approximately every second LTC facility resident who died in 2019 was already offered these ACP consultations. These consultations might support residents’ relatives and healthcare providers in the decision-making process and reduced hospitalization [31, 32]. Nevertheless, our data indicate that fewer in-hospital deaths are associated with ACP consultations. Therefore, ACP consultations should be further supported and offered more frequently. In recent reviews [18, 23], as well as in a national survey of nursing home residents [8], the proportion of residents who reported a desire to discuss their wishes and preferences for care at the end of life ranged from just 60–95%. Other studies also showed that not all residents want ACP and residents trust relatives and staff to make important decisions for them [9]. In addition, residents often already bring advance care documents with them when they move into a facility, so that there is no need for them to speak about ACP [23]. With 50% ADs deposited, our percentage is well above the results of other current surveys on ADs in general with 44% [33] and in nursing home residents with 12% and 33% [34, 35]. Since special trained staff offered the ACP consultation [7], we assume that refusals were not due to a lack of conversational skills. The present finding of a correlation between the number of staff members with PC training and the number of ACP consultations may justify the further PC training of staff members at the investigated LTC facilities.

In total, 14% of the deceased residents in the investigated LTC facilities were accompanied by volunteer hospice services. This is comparable to data from a survey of relatives of deceased residents, who reported companionship by hospice volunteers in about 13% [36]. The data also suggest that residents without relatives are more likely to receive volunteer hospice services. In this context, hospice services may also step in to provide additional support, especially for relief and grief processing for relatives [37].

Across the investigated LTC facilities, there were differences in the expression of surveyed PC indicators. Some studies have shown that the size and location of LTC facilities can impact the level of care provided [19]. Our data show no correlation between facility size and the investigated PC indicators. Nevertheless, facility location is found to be relevant. Only the support provided by voluntary hospice services is more often in urban facilities. It may be that residents in rural LTC-facilities are less lonely because the community cohesion in rural areas is even stronger. It might also be possibly that the density of hospice services is much higher and distances are shorter in metropolitan areas.

Although a recent review showed only few effects of staff training on PC [25], our data show that there seems to be a correlation between the numbers of PC trained staff and certain PC indicators, like support by specialized PC teams and volunteer hospice services, number of hospitalization and ACP consultation. It is possible that the number of staff with a basic PC training partly explains the differences in and the relatively positive results of the key indicators examined in the facilities.

### Strengths and limitations

The present study referred to recent nursing data on PC with respect to a large sample of deceased residents at LTC facilities run by a specific non-profit provider in a single federal state of Germany; thus, the generalizability of the results to other providers or regions might be limited. In addition, only in one facility, none of the employees had a PC training. Due to the low response rate during the COVID-19 outbreak in spring 2020, a high degree of positive self-selection bias amongst the facilities must be considered.

The analyzed nursing documentation did not cover all aspects of PC in the LTC facilities (e.g. it did not include data on special end-of-life rituals). Additionally, not all documented aspects were surveyed (e.g. pain assessment and management). In the analysis of the data, only selected characteristics of the LTC facilities were recorded. Thus, other possible conditioning factors were not analyzed in more detail.

## Conclusion

Overall, a rather positive picture of PC in the participating LTC facilities emerged, although there were differences in the expression of certain indicators between facilities. Measured quality indicators for PC – in particular ACP consultation, volunteer hospice services, and hospital admissions at the end of life – appeared to be superior in LTC facilities with more trained PC staff. Therefore PC training for staff should be promoted. As the results refer to only nursing documentation, the views of relevant stakeholders should also be investigated.

## Data Availability

The datasets generated during the current study are not publicly available due our IRB does not permit the data to be shared publicly. Raw data may be obtained from the corresponding author on reasonable request. All data supporting the findings in this study are included within this published article.

## Abbreviations

ACP: Advance care planning
AD: Advance directives
DNR: Do not resuscitate
HA: Hospital admission
HCP: Health care proxy
h: Hour
ImPAct: Implementation of palliative competence and hospice culture in LTC facilities
LTC: Long-term care
M: Mean
n: Sample size
p: Significance
PC: Palliative care
PoD: Place of death
SD: Standard deviation
SPC: Specialized palliative care
VHS: Voluntary hospice services
